# Prospects for sociogenomics in avian cooperative breeding and parental care

**DOI:** 10.1093/cz/zoz057

**Published:** 2019-12-04

**Authors:** Flavia Termignoni-Garcia, Matthew I M Louder, Christopher N Balakrishnan, Lauren O’Connell, Scott V Edwards

**Affiliations:** 1 Department of Organismic and Evolutionary Biology, Harvard University, Cambridge, MA 02138, USA; 2 Museum of Comparative Zoology, Harvard University, Cambridge, MA 02138, USA; 3 International Research Center for Neurointelligence, The University of Tokyo, Hongo, Bunkyo-ku, Tokyo 113-0033, Japan; 4 Department of Biology, East Carolina University, Greenville, NC 27858, USA; 5 Department of Biology, Stanford University, Stanford, CA 94305, USA

**Keywords:** ATAC-seq, behavior, convergent evolution, epigenetics, neurobiology, phylogeny

## Abstract

For the last 40 years, the study of cooperative breeding (CB) in birds has proceeded primarily in the context of discovering the ecological, geographical, and behavioral drivers of helping. The advent of molecular tools in the early 1990s assisted in clarifying the relatedness of helpers to those helped, in some cases, confirming predictions of kin selection theory. Methods for genome-wide analysis of sequence variation, gene expression, and epigenetics promise to add new dimensions to our understanding of avian CB, primarily in the area of molecular and developmental correlates of delayed breeding and dispersal, as well as the ontogeny of achieving parental status in nature. Here, we outline key ways in which modern -omics approaches, in particular genome sequencing, transcriptomics, and epigenetic profiling such as ATAC-seq, can be used to add a new level of analysis of avian CB. Building on recent and ongoing studies of avian social behavior and sociogenomics, we review how high-throughput sequencing of a focal species or clade can provide a robust foundation for downstream, context-dependent destructive and non-destructive sampling of specific tissues or physiological states in the field for analysis of gene expression and epigenetics. -Omics approaches have the potential to inform not only studies of the diversification of CB over evolutionary time, but real-time analyses of behavioral interactions in the field or lab. Sociogenomics of birds represents a new branch in the network of methods used to study CB, and can help clarify ways in which the different levels of analysis of CB ultimately interact in novel and unexpected ways.

## Introduction

Cooperative breeding (CB) in birds was first described in detail by tropical naturalist Alexander Skutch in 1935. He observed extra-parental behavior, which he defined as non-parental birds assisting breeding by bringing food to fledglings and females in the nest (Skutch 1935). Darwin (1860) and others were already aware of the paradox of explaining the expression of eusocial or cooperative behavior in light of natural selection (Darwin 1860; Hamilton 1964). For both Darwin and Skutch, CB seemed paradoxical. How could natural selection favor individuals who were not the biological parents helping to raise the offspring of others?

Following Skutch’s (1935) early observations, an ecological perspective dominated the study of CB, with a flowering of interest in the late 1970s as sociobiology began to transform behavioral ecology. Accordingly, discussion has fallen largely within one of Tinbergen’s (1963; summarized by Sherman 1988) well-known levels of analysis—the functional consequences of CB. For example, most studies of CB thus far have focused on the adaptive value of CB—why it should be favored in specific environments or demographic contexts (Smith and Price 1973; Brown 1987; Rubenstein et al. 2008). First-generation discussions of the ecology and adaptive value of CB focused on a variety of processes, including the “ecological constraints” model, benefits of inclusive fitness via kin selection, and the tendency for CB to arise in stable habitats (Brown 1987; Reed and Walters 1996; Václav 2000; Cockburn et al. 2008). Long-term studies of cooperatively breeding birds were an important component of this approach and helped categorize CB species as “obligate” or “facultative” (Beck et al. 2008; Li and Merilä 2010; Griesser and Suzuki 2016) with “plural” or “singular” reproductive couples (Brown 1987) among other gradations. In-depth studies of individual species have detected important relationships between the expression of CB and demography (Canestrari et al. 2012) or territory quality (Baglione et al. 2006).

Building on early explorations of the comparative method (Edwards and Naeem 1993; Cockburn 1996), second-generation analyses of the ecology of CB have leveraged global, phylogenetically broad data sets in combination with detailed climatic and environmental data in an attempt to discern ecological covariates of the phylogenetic distribution of CB (Rubenstein and Lovette 2007). The use of phylogenies to study the taxonomic distribution of CB, although questioned early on, is now an indispensable tool for studying environmental correlates of CB and has helped clarify the distinction between the current environments in which cooperative breeders now live and the environments in which CB presumably originally evolved (Ekman and Ericson 2006; Berg et al. 2012). Comparative phylogenetic approaches have detected important relationships between the expression of CB, climatic variation, and other environmental factors (Rubenstein and Lovette 2007; Jetz and Rubenstein 2011). Collectively, these studies have explored the evolutionary origins of CB, another of Tinbergen’s levels of analysis, and point to climatically variable habitats as facilitators of CB, and family groups as an intermediate step between solitary and CB (Griesser et al. 2017).

Although the ecological function and evolutionary origin of CB have been explored for decades with varying degrees of success (Koenig 2017), Tinbergen’s 2 other levels of analysis—ontogenetic processes and mechanisms—have rarely been applied to CB. Here, we focus on those levels of analyses involving molecular mechanisms during development and evolution of CB in birds in the era of genomics, transcriptomics, and other transformative molecular techniques, and provide a roadmap for genome-wide interrogation of the molecular basis of CB in birds. Such molecular explanations of CB should not compete directly with those provided by ecology, which primarily focus on the adaptive value of CB. At the same time, however, a greater understanding of the molecular mechanisms facilitating CB could have profound consequences for our understanding of the plasticity and “evolvability” of CB in different avian lineages and of the extent to which it is adaptive in a current environment.

In the same way that ecological studies identify potential behavioral targets of investigation with -omics methods; -omics methods can greatly clarify potential constraints that could mold the expression of CB and its ability to change when the physical or social environment changes (Kasper et al. 2017a; Rubenstein et al. 2019). For example, the phenotypic plasticity involved in CB can be investigating with -omics methods by analyzing the inter-individual transcriptional and epigenetic variability in changing environments and/or associated to degrees of CB. Birds have nucleated red blood cells which are a rich source of DNA and RNA for comparative epigenomics, genomics, and transcriptomics and offer a unique opportunity to approach sociogenomics of CB in wild populations. Specifically, longitudinal studies of ontogeny in CB species using blood transcriptome and epigenome could reveal developmental constraints molding the expression of CB. Thus, -omics techniques offer an outstanding approach for the study of CB and open new roads into understanding the developmental processes and mechanisms underlying CB.

**Figure 1.**
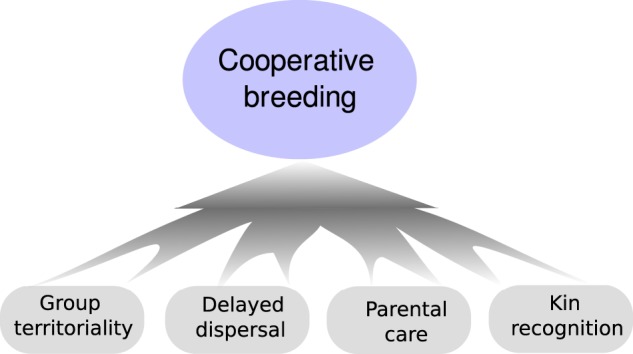
Cooperative breeding perceived as an emergent property from the interactions of individual behaviors such as parental care, delay dispersal, territoriality, and kin recognition.

CB can be considered an emergent property emanating from the interaction of individual behaviors, such as delayed dispersal, territoriality, kin recognition, and parental care (Figure 1). We explore below these individual behaviors, which are natural candidates for molecular interrogation and have been the focus of endocrine and molecular studies, primarily through candidate gene or hormonal approaches (Schmidt et al. 1991; Schoech et al. 1996; Pinxten et al. 2007; Rubenstein et al. 2008; Duval and Goymann 2011; O’Connell et al. 2012). These studies, although likely biased by their directed examination of only candidate substrates for these behaviors, nonetheless provide the foundation for modern studies of avian CB through unbiased genome- and transcriptome-wide approaches. We believe that -omics era approaches to CB will lead to an integrated explanation of the expression of CB as an emergent and complex trait, and, with appropriate comparisons between species, will help provide a framework for understanding why and how CB occurs so frequently in some avian groups and in specific environments (Edwards and Naeem 1993; Cockburn 1996).

## Behavioral Modules Comprising CB

**Figure 2.**
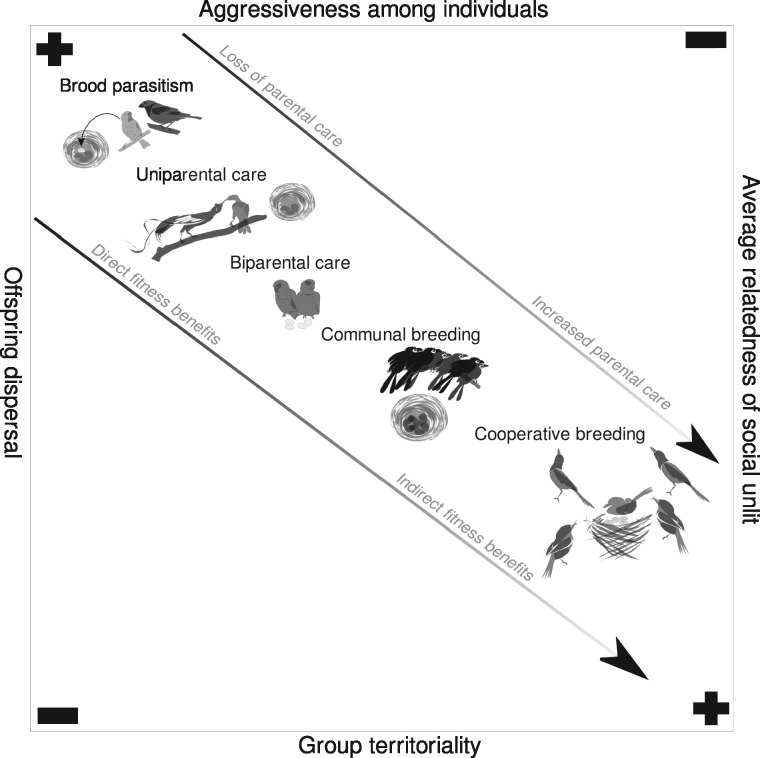
A simplified linear perspective of some features and individual behaviors. Brood parasites are on the other extreme end of parental and cooperative behaviors, with a loss parental care, whereas cooperative breeders display gains in quantity and quality of extra parental care. Plusses and minuses indicate increases or decreases in the variables listed on the axes. The example species used are: brood parasite: cowbirds *Molothrus aeneus*; Uniparental: lesser bird-of-paradise *Paradisaea minor*; bi-parental: parrots; communal breeding: anis (*Crotophaga* sp.); cooperative breeding: a New World jay such as the Yucatan jay *Cyanocorax yucatanicus*.

Research with both natural and experimental populations of birds has helped identify consistent behavioral, demographic, and ontogenetic features of individuals in CB systems that in turn provide targets for genomic interrogation. Individual behaviors, such as delayed dispersal, territoriality, kin recognition, and parental care, are intrinsic to CB, which thus can be considered an emergent property from the interaction of these individual behaviors (Figures 1 and 2).

### Delayed dispersal

One essential characteristic of CB is the presence of delayed dispersal of juveniles from a social unit (Figure 2; Emlen 1995; Ekman 2007). Dispersal determines the extent of gene flow among groups and populations, and gene flow shapes kinship. Thus, dispersal patterns build up—at the population level—the genetic structure and kinship among individuals (Hamilton 1964; Ekman et al. 2004; Mullon et al. 2018). Additionally, population structure and kinship are most likely to be determined by the sex that exhibits cooperation (Hatchwell 2009), and in turn, these patterns influence the evolution of cooperation or aggression (Hamilton 1964; West Sa Griffin and Gardner 2007).

Delayed dispersal is essential for the development of CB behavior, and is crucial to the formation of families and groups (Emlen 1995; Koenig and Dickinson 2004). In some cases, delayed dispersal can facilitate the formation of kin groups; however, there is evidence that some bird species with delayed dispersal and family-living arrangements, such as white-breasted mesites (*Mesitornis variegate*; Gamero et al. 2019), among other species (Hatchwell 2010; Riehl 2013), do not express CB. In fact, adult intolerance to older juveniles hampers the adoption of a cooperative lifestyle, suggesting that family-living and delayed dispersal alone are not sufficient for the evolution of CB (Brown 1974; Ekman et al. 2004; Ekman and Ericson 2006; Gamero et al. 2019).

One recent argument holds that dispersal and social behavior co-evolve, producing two social morphs: a sessile morph which increases the reproduction of relatives from a specific social group and a dispersive morph which increases its own reproduction (Mullon et al. 2018). Simulations suggest that these morphs are caused by linkages between the loci responsible for dispersal and those responsible for social behavior, perhaps via a supergene or similar genetic architecture (Mullon et al. 2018; Rubenstein et al. 2019). However, genomic analyses, such as comparative genomics, genome-wide association studies, and gene expression profiling, are needed to uncover the genetic architecture of CB and elucidate the contribution of coding versus regulatory variation to the expression of CB in birds.

### Territoriality

Territoriality merits special attention due to two key facts. First, territoriality can influence dispersal (Figure 2). Defending a territory year-round for group-living birds depends on the quality of the territory and promotes philopatry (Komdeur 1992; Baglione et al. 2005). Second, an endocrine control mechanism is likely involved when territoriality can be partially explained by testosterone levels in tropical versus temperate birds. Recently, researchers have connected birds that practice seasonal territoriality with higher levels of testosterone compared with birds that practice year-round territoriality (Goymann et al. 2004). Testosterone is a steroid hormone that influences social and aggressive behavior and may be crucial in the expression of CB when social conflicts need to be mediated and breeding adults expresses tolerance to juveniles from the social unit (Soares et al. 2010), known as territorial permissiveness. A recent study of testosterone levels in CB birds across breeding roles revealed a natural variation in levels of testosterone, with low levels during parental care in all members and higher levels in the breeding male than in male helpers during incubation (Pikus et al. 2018), confirming ealier studies (Beletsky et al. 1992). Although testosterone is involved in territoriality and aggression, multiple-related hormones may also play a role in social interactions (Adreani et al. 2018). In many ways, hormones were the first physiological tools used to understand the molecular mechanisms underlying CB (Haig et al. 1994), and the regulatory pathways of endocrine and neuroendocrine hormones need to be explored in more depth. Comparative transcriptomic studies of blood plasma, blood, and brain will shine a light on the hormonal basis of key characteristics of CB in birds (see below).

### Kin recognition

Another example of an individual behavior facilitating CB is parental and conspecific recognition, which has been hypothesized to require an identifiable phenotype (Komdeur and Hatchwell 1999), as well as the ability to learn associatively and match phenotypes, either at the phenotypic or molecular levels. Kin recognition is likely essential for the development of CB via the advantages incurred through indirect effects of kinship—an important component of most but not all CB systems in birds.

Recent work emphasizes the role of multimodal signals in mate choice contexts (Hebets 2011, reviewed in Lindsay et al. 2019) and the same should be true of kin recognition. In some cases, recognition of kin in CB birds is based on experience, rather than genetically encoded recognition signals (Riehl et al. 2015). The bulk of work on learning in birds focuses on auditory signaling (reviewed in Louder et al. 2019; Lynch et al. 2019); and indeed, CB birds such as the long-tailed tits *Aegithalos caudatus* use learned vocal signals for kin recognition (Sharp et al. 2005). Non-CB birds such as Humboldt penguins *Spheniscus humboldti*, storm petrels *Hydrobates pelagicus*, and chickadees (*Poecile* sp.) use odor to recognize related conspecifics or family members (Bonadonna and Nevitt 2004; Coffin et al. 2011; Bonadonna and Sanz-Aguilar 2012; Huynh and Rice 2019). Little research has been conducted in birds to detect multimodal signals in kin recognition. In CB fishes, chemical cues contribute more than visual cues to stimulate the fish to identify relatives (Le Vin et al. 2010). Both odor and visual cues may also be important in recognizing kin suggesting genetically encoded signals. For example, a remarkable age-related phenotype has been hypothesized to be a key factor in the expression of CB in the New World jays, playing an important role in determining specific activities for each age and enabling newborns to recognize members of the group and also to be recognized by the non-breeding helpers (Peterson 1991). The Florida scrub-jay *Aphelocoma coerulescens* and the Yucatan jay *Cyanocorax yucatanicus*, both CB species, have age-related phenotypes that may develop by delaying molting and going through a series of molt transitions for up to 4 years (Hardy 1974; Raitt and Hardy 1976; Peterson 1991; Schoech et al. 1996). Despite many examples of visual cues involved in kin recognition, no studies have been published examining the molecular mechanism of visual cues and kin recognition in birds. Although extremely challenging, understanding systems-level changes in signal properties and receiver physiology in association with kin recognition will provide important insights (Hebets et al. 2016).

Genetic loci with roles in kin recognition or mate choice are logical places to begin a search for molecular mechanisms mediating kin recognition. The major histocompatibility complex (MHC) is a set of immune genes with roles in disease resistance (García Castañeda and León 2013), kin recognition, and mate choice in vertebrates (Manning et al. 1992; Ehman and Scott 2001), but has been investigated surprisingly little in the context of CB in birds (Zelano and Edwards 2002). In mammals, major urinary proteins (MUPs) have also been hypothesized to mediate kin-related behaviors but are likely only relevant to mammals (Ehman and Scott 2001; Hurst et al. 2001; Green et al. 2015; Roberts et al. 2018). The scarcity of loci hypothesized to mediate kin behaviors in birds is a major roadblock to advances in this area and call for comparative transcriptomics and genomics between CB and non-CB species for elucidating the molecular basis of kin recognition in CB systems in birds. Other cognitive behaviors, such as social intelligence skills like intragroup awareness (Pardo et al. 2018) also warrant molecular investigation.

### Uni-parental and bi-parental modes of parental care

Almost all bird species provide some type of parental care, including female uniparental care (8% of avian species), male uniparental care (1% of avian species), CB with more than 2 related individuals caring for young in the same nest (9% of avian species), and biparental care (roughly 80% of avian species; Cockburn 2006). The ecological drivers of evolutionary transitions between these states are not entirely clear. However, there is a rich literature in behavioral ecology regarding sex roles in avian parenting that suggests diversity in parental care strategies is associated with the intensity of sexual selection (e.g., extra pair paternity and frequency of male polygamy) and the social environment (e.g., sex ratio of the population), rather than a relation to gametic size as theorized in most taxa (Liker et al. 2015). For example, in shorebirds, the most common transition sequence is from paternal care to biparental or maternal care, suggesting that sharing parental duties such as incubation or provisioning facilitates the evolution of biparental care and/or abandonment by males (Székely and Reynolds 1995). There is also a large literature on plasticity in provisioning strategies based on environmental or social cues (Ydenberg 1994; Gardner and Smiseth 2011). For example, out of the thousands of bird species with altricial young that need food provisioning by parents, only 7% are uniparental, suggesting sharing the duties of food provisioning is important to maintaining biparental care (Cockburn 2006). Although this diversity in avian reproductive strategies allows for comparative approaches to study the neural and molecular basis of parenting, very little work has been done in this area.

### Brood parasites as a paradigm for the study of parental care

At the other end of the parental care spectrum are brood parasitic species (Figure 2). Brood parasitism, which like CB can be either obligate or facultative, is a behavior in which females lay their eggs in the nests of other birds, forgoing parental care. Brood parasitic behavior has evolved in diverse lineages including insects, fish, and birds (Davies 2000). Facultative brood parasitism is almost exclusively found among precocial bird species and can either target conspecifics or heterospecifics. In contrast, obligate brood parasitism is almost exclusively found among altricial species, highlighting the costs of parental care as an important factor in shaping these evolutionary outcomes (Rohwer and Freeman 1989). Whereas facultative conspecific brood parasitism (CBP) has been considered a precursor to obligate brood parasitism, CBP has also been considered a precursor to CB (Vehrencamp and Quinn 2004) and/or an alternative reproductive tactic to CB. As in CB, kin selection appears to be important in CBP with parasites often laying their eggs in the nests of close relatives (reviewed in Zink and Lyon 2016).

Obligate CB can perhaps be thought of as a gain of additional parental care behavior, whereas obligate brood parasitism might be considered a loss of parental care (Figure 2). A particularly interesting, yet unanswered question is whether the patterns of genomic change associated with gains of complex traits like parental care are similar to scenarios of the evolutionary loss of such traits. One could envision a scenario where both gains and losses of parental care behavior are associated with accelerated evolution in parental care-related genes. Under trait gain, this could involve positive selection, and under trait loss this could reflect the loss of selective constraint. Phylogenetic methods now purport to be able to distinguish these scenarios based on the patterns of nucleotide change between species (Niemiller et al. 2013; Wertheim et al. 2015; Calderoni et al. 2016). As opposed to a discrete trait gain, both brood parasitism and CB can also be considered a shift in the relative timing of reproductive and parental care behaviors.

### Communal behavior

The advent of color-banding birds during the early 20th century led to increased understanding of the biology of communal birds (Brown 1987). Color-banded birds allow for the identification of social units and distinguishing group-territorial behaviors from congregations to forage or to mob predators. Distinguishing social systems from group-territorial behaviors or congregations requires researchers to untangle the composition of age classes and sexes in the social unit and their variation over particular territories. The major characteristics of communal breeding are group territoriality and delayed dispersal. Delayed dispersal promotes composition to establish and to maintain across time and space, which in turn results in group territoriality (Figure 2; Brown 1987; Canestrari et al. 2012).

Communal breeding in birds can take place with or without helpers and be found within many mating systems, such as monogamy, polyandry, or polygyny, with joint nesting or separate nesting. Non-breeding helpers are not always present to cooperate in taking care of the brood during the breeding season (Emlen 1982; Brown 1987) and it is likely that helping behavior evolved after the evolution of communal breeding (Emlen 1982) and/or family living (Koenig 2017; Barve et al. 2019). In addition, at least 3 types of kin selection may be present in communal breeding birds: the nuclear family, sharing families (Brown 1987), or non-relatives (Hatchwell 2010; Riehl 2013), making the genetics of communal breeding more complex than is recognized.

## Neurogenomic Contributions to Parental Behavior

**Figure 3.**
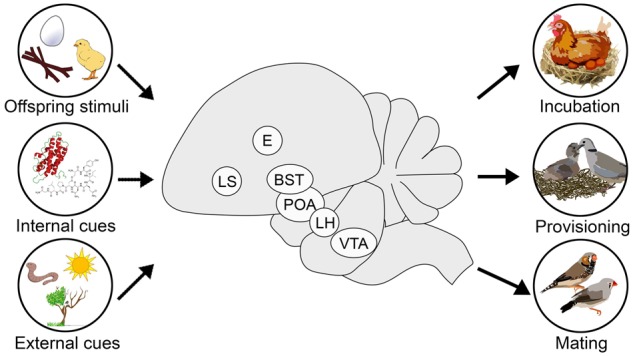
Integration of information from offspring cues, internal physiology, and the environment is performed by core brain regions important for parental behavior in birds, such as promoting egg incubation and offspring provisioning while rejecting mating opportunities. Information on brain regions important for parental behavior incorporated from the following publications: chickens: Chokchaloemwong et al. (2013); ring doves: Buntin et al. (2006); quails: Ruscio and Adkins-Regan (2004); zebra finches: Hall et al. (2014). BST, bed nucleus of the stria terminalis; E, ectostriatum; LH, lateral hypothalamus; LS, lateral septum; POA, preoptic area; VTA, ventral tegmental area.

Parental behavior is produced by a set of interconnected brain regions that integrate both external information about the social environment with internal physiological cues encoding reproductive state and energy stores (Figure 3). The brain regions, neuronal cell types, and genomic patterns associated with parental behavior is an area of increasing research focus, although little is currently known about these associations in birds. Here, we review what is known about the neural and genomic basis of parental behavior in birds, although we note that the diversity of parental care strategies, like cooperative behavior in jays, biparental behavior in songbirds, and loss of maternal care in brood parasites provide fertile ground for future research in this area.

### Brain regions and neuromodulators associated with parental care

Brain regions that regulate social behavior and evaluate the salience of stimuli are conserved across all vertebrates and have been termed the “social decision-making network” (O’Connell and Hofmann 2011). This set of brain regions include the hypothalamus, forebrain regions like the lateral septum and bed nucleus of the stria terminalis, and brain regions associated with dopaminergic signaling like the ventral tegmental area and nucleus accumbens. Although parental behavior has evolved many times independently across animals, many of the same brain regions and neuromodulaters are associated with parental behavior across vertebrates (Dulac et al. 2014; Fischer et al. 2019). With emphasis on birds, most studies investigating the neural basis of parental care focus on nesting behavior in the biparental zebra finch, parent–chick interactions in biparental doves, and maternal care in chickens (Buntin et al. 2006; Chokchaloemwong et al. 2013; Hall et al. 2014). Many of these studies utilize the immediate early gene c-Fos as a proxy of neural activity relating to a behavior of interest compared with a control. These studies are often paired with immunohistochemical colocalization of c-Fos with a neuronal cell-type marker of interest to infer function of specific neuromodulators in parenting behavior.

In zebra finches *Taeniopygia guttata*, nesting behavior shows sex-specific patterns of neural activation. In the bed nucleus of the stria terminalis and the anterior hypothalamus, only females show increases in c-Fos during nesting, which also correlates with time spent in the nest (Hall et al. 2014). Despite this sex-specific increase in c-Fos in the bed nucleus of the stria terminalis, c-*Fos* colocalization with mesotocin (the avian homolog of mammalian oxytocin) is higher in nesting birds in both sexes (Hall et al. 2015). Similarly, c-Fos colocalization increases in arginine vasotocin (the avian homolog of mammalian arginine vasopressin) neurons in the periventricular nucleus of the preoptic area is associated with nest building in female, but not male, zebra finches (Klatt and Goodson 2013). Despite these sex differences, administration of an arginine vasotocin receptor V1a antagonist decreases time in the nest for both males and females (Klatt and Goodson 2013). On the other hand, only male zebra finches have increased neural induction in the dopaminergic cells in the ventral tegmental area, which is correlated with picking up nesting material (Hall et al. 2014, 2015), suggesting nest building is a reinforcing or rewarding behavior for male zebra finches.

In contrast to the sex differences observed in zebra finch nesting behavior, response to chicks after separation shows no sex differences in neutral activity patterns. When reunited with their chicks, both males and females show increase neural activity within the preoptic area, bed nucleus of the stria terminalis, the paraventricular nucleus, and the ventromedial hypothalamus (Figure 3; Fazekas et al. 2019). Interestingly, the number of c-Fos-positive neurons in the nucleus accumbens correlated to the frequency of feeding nestlings, suggesting that in addition to nest building, chick provisioning is also likely a reinforcing behavior in zebra finches. Further studies need to be performed to determine the neuronal cell types involved in zebra finch parental care, but is it clear that similar neuronal mechanisms are likely responsible for the biparental care observed in this species.

Biparental ring doves *Streptopelia risoria* also show few sex differences in neural correlates of parental behavior. For example, in a study examining c-Fos immunoreactivity when parents were presented with their chicks after a separation period, there were no sex differences in the broad patterns of neural activity observed (Buntin et al. 2006), similar to the recent study in zebra finches (Fazekas et al. 2019). When these parental ring doves were reunited with chicks after a separation period, there was increased neural activity in the lateral septum, preoptic area, lateral hypothalamus, and the bed nucleus of the stria terminalis compared with parents that were not reunited with their chicks (Figure 3). Research in ring doves has also tightly linked feeding-related neuronal circuitry to provisioning behavior. Both male and female ring doves provision chicks with crop milk, which coincides with increased feeding behavior (Lehrman 1955). During this time, there is increased c-Fos colocalization in agouti-related peptide (AgRP) and neuropeptide Y (NPY) neurons, which are well-known to increase food intake in vertebrates (Strader and Buntin 2003). Interesting, increased prolactin levels during post-hatchling care drives this increase in AgRP and NPY expression and subsequent feeding behavior (Strader and Buntin 2001). The role of prolactin in promoting parental care seems to be brain region specific, as lesions of the preoptic area inhibit crop feeding to chicks but do not affect prolactin-induced hyperphagia (Slawski and Buntin 1995).

In addition to monitoring neuronal activity with immediate early gene markers, parental behavior also seems to be modulated by changes in cell number in various brain regions. This work has mostly been done in the context of maternal care in native Thai chickens *Gallus domesticus*. A study investigating the involvement of mesotocin in maternal care showed the number of mesotocin neurons decreased in the preoptic area when hens were deprived of their chicks (Chokchaloemwong et al. 2013). Interestingly, when eggs were replaced with chicks, the number of hypothalamic mesotocin cells increased while the number of dopaminerigic cells in the nucleus intramedialis and nucleus mamillaris lateralis decreased (Sinpru et al. 2018). These data suggest that throughout reproductive transitions, like switching from egg incubation to caring for chicks, neuronal activity and total number of cells producing important behavioral neuromodulators can change.

With these limited number of studies, it seems that the bed nucleus of the stria terminalis and preoptic area of the hypothalamus are core nodes in the network of brain regions that regulate parental behavior in birds. Other brain regions and neuromodulators that regulate parental behavior seem to differ by species and specific parental strategy. More comparative data across species are needed to identify commonalities across birds and how these neuronal mechanisms may be evolutionarily tuned across species and sexes. Additionally, we recommend that as studies expand across more species, more untargeted methods like whole brain imaging of neural activity and molecular profiling of active neurons using transcriptomics should be used to investigate the neural mechanisms of parental care beyond the hypothalamus and mesotocin.

### Neurogenomics of parental behavior in birds

Only one transcriptomic study examining the proximate mechanisms of parental behavior in birds has been published to date and focuses on brood parasitism. In this study, Lynch et al. (2019) compared gene expression in the preoptic area of brood parasitic cowbirds and closely related, non-parasitic, adult, and juvenile blackbirds. Intriguingly, they found that among genes differentially expressed between parasitic and non-parasitic species, cowbirds more closely resembled juvenile than adult blackbirds. This pattern suggests that adult brood parasites show neotenic (juvenile-like) gene expression in the preoptic area. Given that in CB species, helpers in a sense delay reproductive maturation, neoteny may be observed in CB as well. While BP species show neoteny in parental care-related regions of the brain (Lynch et al. 2019), CB species may show plasticity and/or neoteny in tissues involved in sexual reproduction and/or secondary sexual characteristics.

Insights from the type of study by Lynch et al. (2019) shed light on the utility of comparative transcriptomics into comparative phylogenomics. The genes found to be differentially expressed in that study are natural targets for comparative phylogenomic studies; by analyzing and exploring these regions of the genome, insights can be gained into the genomic drivers of variation between parental or non-parental species in a clade. Whole reference genomes are more likely to be available than transcriptomes, especially for bird species difficult to collect, making comparative studies at the level of the genome more feasible.

## Research Designs for -Omics Studies of CB and Parental Care in Birds

Species whose behavior has been studied extensively in the wild are ideal for comparative analysis examining the evolution and mechanisms underlying reproductive behavior. Studying ecologically relevant behaviors in the wild is challenging, but the ability to pair behavior with information on molecular mechanisms and fitness consequences can be especially transformative. Moreover, utilizing well-studied species in their natural habitat avoids misclassifying CB species (Griesser and Suzuki 2016). Despite the strengths of these natural avian systems, not all species are appropriate for all types of -omics approaches, especially when animals need to be euthanized for tissue sampling. For some questions involving the molecular correlates of CB, tissue-specific collection and gene expression analyses may be required. However, it may be possible to employ non-destructive techniques, such as transcriptomics of peripheral blood, to identify how individuals perceive each other (Louder et al. 2018). Depending on the questions being asked and the logistics of accessing and observing individuals in the field, some -omics approaches may be more appropriate or feasible than others for studying reproductive behaviors in the laboratory versus the wild.

### The -omics of avian CB in the laboratory and field

Long-term field studies are well-known for their ability to capture the complexity of traits in wild populations. However, it is only recently that such studies have been complemented with -omics technology to produce new insights into genetic and behavioral mechanisms. Some of the best examples of this dual approach come from birds, with new insights into inbreeding and migration (Chen et al. 2016, 2019), speciation (Lamichhaney et al. 2018), microevolutionary change (Bosse et al. 2017), and the fitness effects of pathogens (Asghar et al. 2015). Critical to the success of such endeavors is collecting samples appropriate for -omics technologies throughout the duration of the field studies. An excellent study system for this integrative approach is the Florida scrub-jay (*A.* *coerulescens*), for which there is an extensive long-term pedigree and blood samples suitable for genetic analysis (Chen et al. 2016). However, because this species has a vulnerable conservation status, only non-destructive samples are accessible and a combination of transcriptomics of peripheral blood, longitudinal studies of ontogeny, and comparative genomic are promising analyses for the study of CB.

**Figure 4.**
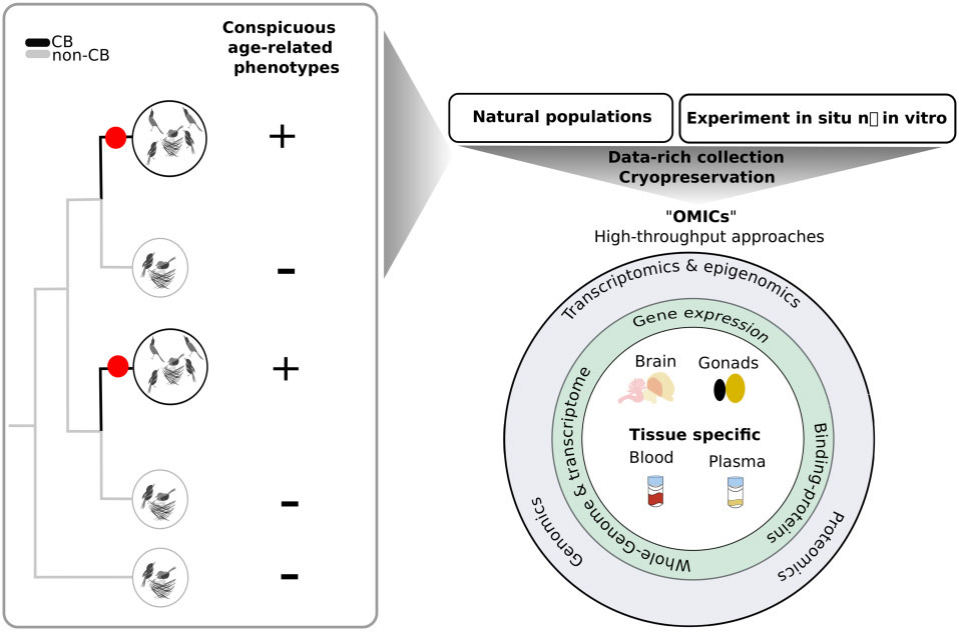
Comparative approaches to cooperative breeding can guide sampling for -omics analysis. *Left*, a phylogenetic tree with 2 pairs of closely related but convergently evolved cooperative breeders, illustrating the comparative approach. Species with age-related phenotypes involved in kin recognition are indicated. The convergent origins of CB are indicated with red circles. *Right*, sources of macromolecules and epigenetic states useful in the study of cooperative breeding, sampled from natural populations or from experiments in the wild or in the laboratory. Specific tissues of relevance to cooperative breeding and used to extract different types of macromolecules are indicated.

There are three general approaches to integrating experimental -omics approaches in social and parental behavior in vertebrates: (1) species from the wild are moved to the lab to isolate environmental effects and distinguish them from genetic effects (Kasper et al. 2017a, 2018; Fischer et al. 2019; Lynch et al. 2019); (2) experiments can manipulate ecological conditions or social contexts in the wild (Baglione et al. 2006; Canestrari et al. 2008); (3) and collection of samples in the field can be used to analyze molecular signatures at the time of capture (Fischer et al. 2019). For such approaches, samples such as blood and plasma can be collected non-lethally, or cryo-preserved tissues of various organs can be obtained after euthanasia (Figure 4). The success of such approaches depends critically on the quality of the behavioral observations associated with specimens taken and the degree to which the natural history is understood.

The next best strategy to integrate behaviors in the wild with an -omics approach is behavioral studies in the laboratory. Such studies have been used extensively to understand mechanisms of behavior in vertebrates, such as parental care (O’Connell et al. 2012; Bedford and Hoekstra 2015; Smiley and Adkins-Regan 2016; Bendesky et al. 2017), aggressiveness (Ruploh et al. 2013), and cooperation (Soares et al. 2017). These studies address the extent of adaptive behavioral plasticity by controlling for genetic background and environmental conditions. Because behavior is the cumulative output of multiple levels of biological organization (genes, hormones, neural circuits, and individuals and social units), both laboratory and field studies are useful in examining proximate and ultimate mechanisms.

### Comparative approaches and convergent evolution as a framework for studying avian sociogenomics

Comparative genomics offers an important avenue for understanding the genetic basis of CB in birds. Most examples of the genetic basis of social and parental behavior in animals have been discovered at least in part through laboratory crosses, experimental manipulations, or genetic characterization and comparison of inbred strains (Bendesky et al. 2017; Kohl et al. 2018). Some organisms known for their diversity and behavior in the wild, such as poison frogs, are amenable to experimental analysis in the laboratory. However, because most cooperatively breeding birds are unlikely to function or even survive well in a laboratory or an aviary setting, non-manipulative, comparative approaches are likely to be crucial for furthering our understanding the sociogenomics of CB in birds (Lamichhaney et al. 2019).

Comparative analyses of discrete and continuous traits on phylogenetic trees have become quite sophisticated in recent years, with diverse models describing how traits evolve on trees, how natural selection on traits can be detected, and how changes in traits, such as behavior, can be associated with changes in multiple variables (Ord and Martins 2006; Hultgren and Stachowicz 2009; Clavel and Morlon 2017; Forthman and Weirauch 2018; Gerson et al. 2019). Such analyses rely on gathering trait data from a large number of species and mapping traits onto a phylogeny to identify which trait or traits change state multiple times across the phylogeny.

As recognized long ago by the founders of comparative methods (Felsenstein 1985; Harvey and Pagel 1991), multiple origins of a trait on a tree are often required to associate that trait with a second variable, such as changes in the environment. Multiple changes are required because it is sometimes indefensible to conclude that a behavior is associated with a second variable when that behavior has only arisen once in a single lineage. In such a situation, the behavior could be associated with the second variable simply by chance, especially on long branches when many coincident changes in many traits could accrue by chance, although such unique natural experiments can sometimes be extremely compelling, such as examples of unusual but unique evolutionary signals along the human lineage (McLean et al. 2011). From a statistical standpoint we are often hard pressed to explain the origins of traits that have evolved on a single lineage within a clade. As a result, investigating traits with multiple origins arising across a large sample of species, also known as convergent evolution, is now considered the most robust design for comparative studies.

For these reasons, convergent evolution has become a key focus in the effort to link genomic change with phenotypic traits in animals (Fischer et al. 2019; Lamichhaney et al. 2019; Rubin et al. 2019; Sackton et al. 2019). In the last 10 years, great progress has been made in understanding the contribution of specific genes, regulatory regions, or gene networks to phenotypic traits exclusively through comparative analysis of convergently evolved traits. These discoveries are often made on clades that are poor experimental models, either because of their intractability in the lab or the difficulty of acquiring live samples from the wild. In addition, these studies often start with a foundation of whole genome or whole transcriptome studies from a suite of species in the clade and for this reason can be daunting to undertake (Zhen et al. 2012; Kapheim et al. 2015; Sackton et al. 2019). Such fruitful collaborations between biologists with an expertise in the diversity of phenotypes in the clade and computational biologists with expertise in analyzing large, complex -omics data sets have shown that such studies can be transformative.

A variety of recent examples from birds exploit convergent evolution to understand the origins of complex phenotypic traits, such as loss of flight (Sackton et al. 2019), vocal learning (Pfenning et al. 2014), and adaptation to high altitudes (Projecto-Garcia et al. 2013; Natarajan et al. 2016). Many of the species in these studies are not traditional models for laboratory experimentation. However, comparative studies of convergent social behavior in animals have made the most progress in the social Hymenoptera, with several recent studies providing compelling examples of phylogeny-enabled discovery of the genetic networks underlying social behavior (Woodard et al. 2011; Kapheim et al. 2015). Comparative analyses of the ecology of CB in birds have made important progress through the use of large-scale phylogenies (Jetz and Rubenstein 2011), but thus far such approaches have not addressed the genetic basis of CB. Groups of birds such as New World jays, in which CB has arisen multiple times and in which CB appears comparable and consistent across lineages, are ripe targets for genomic study of CB through convergent evolution.

### Phylogenetic methods for genomic discovery in CB

Several phylogenetic and statistical approaches can facilitate our understanding of the genomic sources of CB, especially under a scenario of convergent evolution (Figure 4). Comparisons of patterns of gene expression between cooperative and non-cooperative species, ideally closely related, are a powerful means of discovering genomic sources of CB. Similar approaches have facilitated discovery of genes and gene networks underlying sociality in Hymenoptera (Woodard et al. 2011; Kapheim et al. 2015). However, care should be taken when analyzing transcriptome data in a comparative context. It is imperative that gene expression data be treated as one would treat any continuous character compared between species—with appropriate comparative methods designed with phylogenies in mind (Rohlfs and Nielsen 2015; Guang et al. 2016; Dunn et al. 2018). Failure to account for phylogenetic relationships when analyzing gene expression data can lead to unfounded inferences and false positives and negatives (Dunn et al. 2018).

Additionally, efforts to measure gene expression differences between species or life history stages are very sensitive to a variety of pitfalls, including presence of paralogs, biases in mapping to reference genomes, and other complications such as incorrect estimation of the effective length of transcripts and genes (Romero et al. 2012; Freedman et al. 2019). Moreover, it is generally agreed that accurate measurement of gene expression within and between species requires at least one closely related reference genome (Freedman et al. 2019). Previously, one of the challenges with doing -omics studies in wild populations was dearth of reference genomes, where the few reference genomes available were from distantly related species. Reference genomes are becoming easier to assemble, and, although long-read and linked-read sequencing technologies are still in their infancy in their application to birds, they have made the production of high-quality reference genomes for a variety of species much more practical (Gordon et al. 2016; O’Connor et al. 2019). Still, comparative transcriptome studies without reference genomes have proven their worth by revealing a variety of exciting new leads for the study of the molecular basis of sociality in animals. We are quickly approaching the time when producing a reference genome for each species from which transcriptomes will be assembled is feasible, and doing so will improve the utility of comparative genomic and transcriptomic analyses for understanding the molecular basis of CB.

Examining patterns of positive selection and relaxed constraint across the genome offers another means of identifying candidate loci for CB in birds. Detecting both positive selection, in which natural selection accelerates the evolution of a gene or regulatory region in a target species, and relaxed selection, in which a genomic region might accelerate due to removal of strong purifying selection along a particular lineage, are key approaches for studying the comparative genomics of CB. Additionally, the evolutionary rate of formerly neutral regions can decrease and become constrained, and newly constrained regions of the genome can arise and turnover *de novo* in different clades, suggesting the origin of new genic and regulatory functions not found in other clades (Lowe et al. 2015). There is a long history and a variety of methods for detecting positive selection on protein-coding regions along a phylogeny (reviewed in Barrett and Hoekstra 2011; Rey et al. 2018; Partha et al. 2019). In birds and other groups, such methods have been used with good success to identify genomic regions associated with a variety of phenotypic traits, and genome-wide interrogation of the patterns of selection across species can be useful in identifying strong, consistent targets of selection maintained over evolutionary time (Shultz and Sackton 2019). More recently, changes in the rate of evolution of regulatory regions have allowed researchers to link genomic and phenotypic change across a phylogeny. Conserved, noncoding regions of the genome, such as conserved non-exonic elements (CNEE), are a useful and tractable inroad to the complex landscape of noncoding regulatory function across the Tree of Life. Such elements often have a regulatory function, which can be corroborated by analyses of chromatin state (using methods such as ATAC-seq) and functional assays like enhancer screens (Huang and Ovcharenko 2015; Kvon et al. 2016; Sackton et al. 2019). New statistical methods provide efficient means of identifying where on a phylogeny such regions change their evolutionary rate, signaling a change or loss of function (Hu et al. 2019). Comparative genomic and epigenomic methods like those described above are likely to play an important role in the discovery of genomic substrates for CB.

### New World Jays: a model system for the -omics of CB

The study of New World jays offers ample opportunities for integrating long-term field studies with comparative ’omics approaches to unravel the underlying mechanisms of CB. In the family Corvidae, >50% of species are known to exhibit CB behavior to some extent (Ekman and Ericson 2006; Griesser and Suzuki 2016). For example, CB has been studied for decades under natural conditions in the Yucatan jay and other *Cyanocorax* jays (Hardy 1974, 1979; Raitt and Hardy 1976, 1978; Raitt et al. 1984) as well as *Aphelocoma* jays (Brown 1974; Woolfenden 1984; Schoech et al. 2004; Coulon et al. 2008). Importantly, there are multiple examples of closely related CB and non-CB species within the New World jays, greatly facilitating comparative work and analyses of convergent evolution (Ekman and Ericson 2006; Bonaccorso et al. 2010; Berg et al. 2012). Long-term studies of the Florida scrub-jay, an easily accessible species with abundant field and demographic data, have already provided fruitful for use of genomic approaches to understand fine-scale demography, inbreeding, and natural selection (Chen et al. 2016, 2019; Aguillon et al. 2017).

A down-side of New World jays as a model system is that many of the species live in the tropics, and, despite decades of field study on many of the species, the natural history of some species is still poorly known. For such species, sampling tissues from different ontogenetic stages, which are often easily detectable through changes in bill or iris color, might be a fruitful inroad to begin molecular studies. The developmental level of analysis of CB could be interrogated with blood transcriptome or blood plasma proteome analyses from non-destructive samples in longitudinal studies. Samples from small numbers of known-age individuals, even without detailed behavioral information, could be useful for the study of blood and blood plasma, proteomics, transcriptomics, and genomics. The concentration of binding proteins required to transport relevant hormones, as well hormone profiles themselves, can easily be measured from blood plasma (Schmidt et al. 1991; De la Cruz et al. 2003; Pinxten et al. 2007; Duval and Goymann 2011; Pintér et al. 2011; Brown and Vleck 2017). Such sampling may be the best way to begin the molecular study of CB in species that are challenging to access in the field, but for which comparisons between species can be particularly revealing.

## Functional Characterization of CB: Proteomics, Transcriptomics, and Epigenomics

Complex traits, such as cooperative behavior, require the application of diverse approaches at multiple levels of analysis in order to comprehensively characterize their function (Kasper et al. 2017b, 2018). Recent developments in -omics techniques, such as transcriptomics, genomics, proteomics, and epigenomics, provide robust approaches to identify the candidate mechanisms involved in the phenotype (Figure 4). Manipulative functional genomics techniques, such as pharmacological inhibition or virus-mediated gene manipulation, will one day help reveal causal mechanisms involved in the expression and evolution of the complex trait and are in many cases essential for a thorough understanding of the mechanisms underlying behavior. There are certainly precedents for experimental manipulation of hormones in the field as a means of interrogating life history phases in birds. To our knowledge, manipulation of gene sequences or gene expression has yet to be applied to the study of CB in birds. “Functional” characterization of CB in birds will likely rely for some time on correlations of CB-related behaviors and the expression of transcriptomic or epigenomic traits, such as gene expression or epigenetic markings (Rubenstein et al. 2019).

Identifying genes associated with social behaviors has been greatly facilitated by transcriptional profiling in brain regions regulating social behavior such as the social decision-making network (Horton et al. 2018; Lynch et al. 2019). In addition, comparing transcriptional profiles of key organs, such as gonads and brain, can reveal key factors involved in alternative reproductive phenotypes. For example, in fish it has been suggested that gene networks underly the ability of non-dominant males to mimic females in appearance and behavior in order to gain fertilization and group-spawning (Todd et al. 2018). Combining transcriptome results of relevant organs such as brain and gonads in a comparative framework in the study of CB is a promising approach, but there are few examples, particularly in birds.

Novel and non-invasive approaches to the study of social behavior include the use of blood plasma for proteomics and blood for transcriptomics. Blood plasma contains a highly complex mixture of proteins, which could be studied through proteomic assays such as mass spectrometry or multiple antibody assays. Proteomic assays detect the multiple secreted signaling proteins connecting organs in the organism, such as cytokines or trophic factors (Coppola 2014). For example, the androgen precursors dehydroepiandrosterone (DHEA) which circulates in blood plasma is synthesized in the avian brain (Pradhan et al. 2008) and is thought to drive territoriality and aggression (Landys et al. 2013). Similar results can be acquired with blood transcriptome, revealing the molecular complexity of blood as individuals interact with and perceive each other via signalling molecules reaching the brain (Louder et al. 2018). Blood plasma proteomics and blood transcriptomics have rarely been applied to behavioral questions in vertebrates, but the methods are promising, particularly because they offer a less invasive approach to studying brain function and signaling molecules involved in social behavior and CB. The application of these promising -omics techniques to the study of CB systems would be novel and could uncover relevant molecules and mechanisms underlying this behavior.

Comparative epigenomics could also shed light on the regulation of CB in birds. With the application of -omics technology such as bisulfate sequencing, chromatin immunoprecipitation sequencing (ChIP-seq), and assays for Transposase-Accessible Chromatin-using sequencing (ATAC-seq) (Herman et al. 2017; Letelier et al. 2018), it is possible to screen for active regulatory networks by identifying genomic regions with open chromatin, which are accessible for transcription (Kratochwil and Meyer 2014). However, few studies in birds have focused on the potential epigenetic mechanisms of social behavior thus far. DNA methylation levels in young cooperatively breeding superb starlings *Lamprotornis superbus* revealed potential epigenetic effects on the development of social phenotypes via methylation of the promoter for the glucocorticoid receptor (Rubenstein et al. 2016). In social insects, caste differences are related to a major reduction of DNA methylation (Lyko et al. 2010; Standage et al. 2016). In mammals, an epigenetic mechanism is involved in the expression of mothering style through a caregiving behavior, highlighting this mechanism as a contributor to the experience-induced onset and maintenance of this social behavior (Stolzenberg et al. 2014; Peña and Champagne 2015) Together, these studies suggest the potential role of methylation in social behavior.

In our current work we are studying New World jays such as *Aphelocoma* or *Cyanocorax* and developing longitudinal studies of ontogeny and collecting samples in a field setting from each age-related phenotype from CB and non-CB species, complemented with transcriptomes of blood, brain, and gonads and species-specific annotated reference genomes. A comparative transcriptomic analysis will be conducted and expanded to a comparative phylogenomic analysis to answer questions about how CB has evolved in these clades.

## Challenges and the Future Directions

Distinguishing different forms of CB in birds and the homologies between them is a prerequisite for developing hypotheses for the molecular basis of CB, especially in a comparative framework (Edwards and Naeem 1993, 1994). New avenues of research opened up by the -omics era first and foremost depend on proper interpretation of behavioral modules and behaviors of cooperatively breeding species in their natural environments.

Due to the plasticity and dependence of diverse fundamental brain and behavioral processes, studying the molecular basis of a complex trait such as CB requires the application of diverse -omics techniques on both short and long time-scales. Understanding the details of expression and epigenetic changes associated with changes of behavioral state will require interrogating natural and experimental populations with diverse -omics techniques. Such sampling may well turn out to be a major limiting factor for many studies, since researchers studying bird behavior are often more hesitant to destructively sample individuals, especially those from long-term studies. Additionally, not all techniques are well standardized in wild animals and those approaches requiring euthanasia may be adopted slowly. Approaches such as comparative genomics, blood transcriptomics, and blood plasma proteomics will offer useful opportunities in many scenarios, such as when working with difficult-to-access or threatened species. On the other hand, in less restrictive situations, invasive approaches have already yielded exciting new insights, such as recent work in brain transcriptional profiles studied in birds behaving in the wild (Horton et al. 2019; Lynch et al. 2019).

A phylogenetic framework is essential to understanding the evolution of CB (Edwards and Naeem 1993), but has thus far been applied primarily in ecological settings. Comparative genomics combined with novel statistical approaches incorporating phylogeny can yield useful insights into the evolution of CB in birds, just as it has for other phenotypic traits (Lamichhaney et al. 2019; Sackton et al. 2019). Furthermore, mapping the processes and the mechanisms underlying CB with a whole-transcriptomes and epigenomes in a phylogenetic context offer unique opportunities to understand how social function evolves and diversifies (Kapheim et al. 2015; Kapheim 2018).

Understanding the function and molecular mechanism of CB will help clarify a major topic in evolutionary biology that has intrigued scientists for decades: how can natural selection favor non-breeding individuals who help raise others’ offspring? Some have argued that CB is not an adaptive trait or evolutionarily stable strategy (Smith and Price 1973; Jamieson and Craig 1987) but a functional characterization of CB will help answer this question and will help clarify how such behavior can be inherited and modified through time. Insights from genomics suggest that individual behaviors such as kin recognition or delayed dispersal coevolve with social behavior with the emergence of a social polymorphisms (Mullon et al. 2018). Supergenes or novel genetic architectures can lead to such heritable associations with behavior (Mullon et al. 2018; Rubenstein et al. 2019).

The sociogenomics of social behaviors such as CB and parental care in birds is still in its infancy. The functions, mechanisms, and the genomic architectures of these behaviors are fertile avenues to be understood with high-throughput techniques in combination with data collection in laboratory and field settings. Complex traits like CB act as a conceptual hub that brings together many fields, from neuroscience to genomics and behavioral ecology. Diverse technologies and experimental approaches will be required for a full comprehension of the evolution and mechanisms underlying CB in birds.

## Author Contributions

All authors planned the organization and themes of the manuscript. F.T.-G. led the writing of the manuscript, with extensive contributions from all authors. All authors edited and approved the final manuscript.
